# Urothelial Carcinoma Recurrence in an Ileal Neobladder Nine Years after Primary Surgery with Muir-Torre Syndrome

**DOI:** 10.1155/2016/4762514

**Published:** 2016-03-30

**Authors:** Floryn Cherbanyk, Marc Prod'homme, Edgardo Pezzetta, Bernard Chevaux, Anass Anaye, Jean-Joseph Boillat

**Affiliations:** ^1^Department of Surgery and Urology, Riviera-Chablais Hospital, 1820 Montreux, Switzerland; ^2^Department of Oncology, Riviera-Chablais Hospital, 1800 Vevey, Switzerland; ^3^Department of Radiology, Riviera-Chablais Hospital, 1820 Montreux, Switzerland

## Abstract

We report a patient who presented with a urothelial carcinoma recurrence developed nine years after radical cystoprostatectomy, related to Muir-Torre syndrome.

## 1. Introduction

It is well known that most of urothelial carcinomas are papillary and multifocal and can recur after endoscopic resection. More than 70% of patients develop one or more recurrence after transurethral resection (TUR) of a primary noninvasive bladder tumor [1]. In urinary diversion using isolated gut segment, secondary malignancy as adenocarcinomas resulting of the irritation of the intestinal mucosa by the contact with urine is not uncommon, which has been rarely reported and is a secondary malignancy as urothelial carcinoma.

## 2. Case Presentation

We present a patient with a urothelial carcinoma recurrence developed nine years after primary surgery.

In November 2004, a 66-year-old man had been diagnosed with a grade II to III urothelial carcinoma because of the finding of a unilateral hydronephrosis due to a stenosis by a tumor in the left ureter. His laboratory findings were within normal limits and thoracoabdominal computed tomography did not show evidence of metastatic disease. A radical cystoprostatectomy with W-shaped ileal neobladder was performed. The urothelial carcinoma grade III was located in the area of the opening of left ureter. There was also an in situ carcinoma in the same area and a spread out in the perivesical tissue. No cancer was detected in the prostatic urethra. The TNM classification was pT3a pN1 (1/13) cM0, G3, L1, and stage IV. After the surgery, the patient underwent adjuvant chemotherapy by 3 courses of cisplatin and gemcitabine.

In 2006, he underwent a right enlarged hemicolectomy because of an invasive adenocarcinoma. In 2010, a Muir-Torre syndrome was discovered after a biopsy of sebaceous cysts. The genetic analyses revealed a MSH2 gene mutation (c.293_296dup (p.Val100X) heterozygous duplication). Then, a subtotal colectomy for a local recurrence of the colic adenocarcinoma was performed.

Seven years later in 2012, the patient reported haematuria. A cystoscopic examination with TUR biopsy of the neobladder was performed, showing a malpighian urothelial metaplasia.

**Figure 1:**
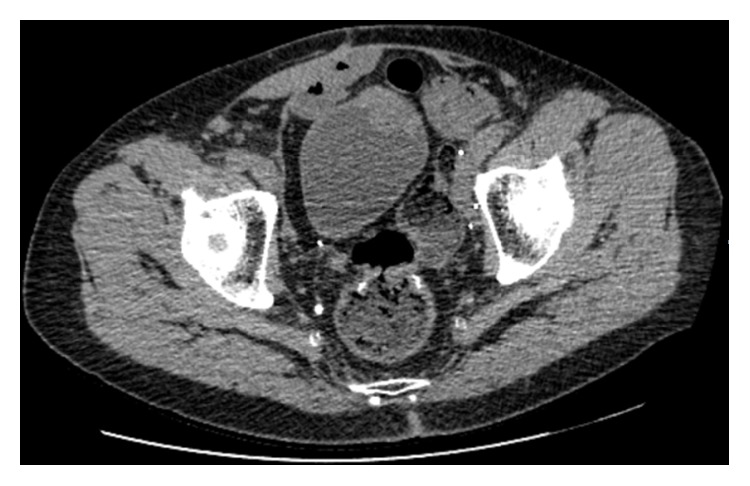
Abdominal CT-scan: lesion of the anterior wall of the neobladder.

In 2014, actually nine years after the ileal neobladder surgery, he presented with haematuria again. Preliminary blood results were within normal limits, apart from anaemia and chronic kidney failure (creatinine clearance 40 mL/min). A native abdominal computed tomography was performed and showed a tissular thickening on the anterior and left parts of the neobladder, dimensions 14 × 8 mm (Figure 1).

**Figure 2:**
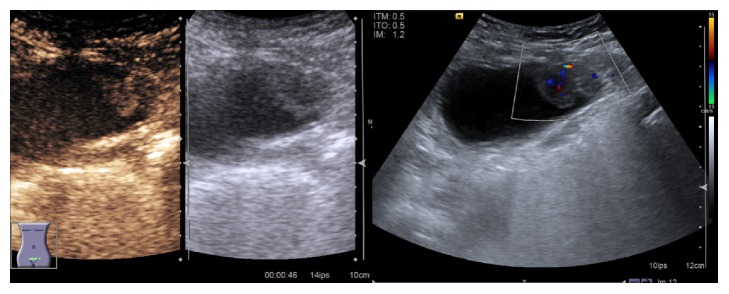
Pelvic US pictures.

The radiological assessment was completed by ultrasonography with intravenous injection of sulphur hexafluoride (SonoVue®) contrast agent (Figure 2). It showed enhancement of the mass during arterial and portal phases, with a wash-out in late venous phase.

The cystoscopic view identified a local tumor, anterior and on the left part of the neobladder. We performed a TUR complete resection of this lesion.

**Figure 3:**
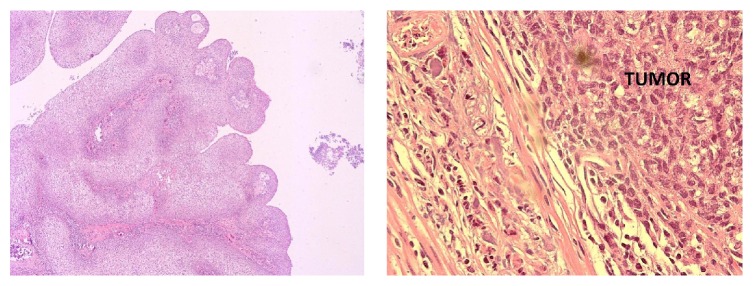
Histologic cross sections showed a high grade papillary urothelial carcinoma with muscularis propria invasion.

The histologic analysis revealed a high grade papillary urothelial carcinoma with a malpighian differentiation. This tumor invaded the muscularis propria of the ileal neobladder (Figure 3).

**Figure 4:**
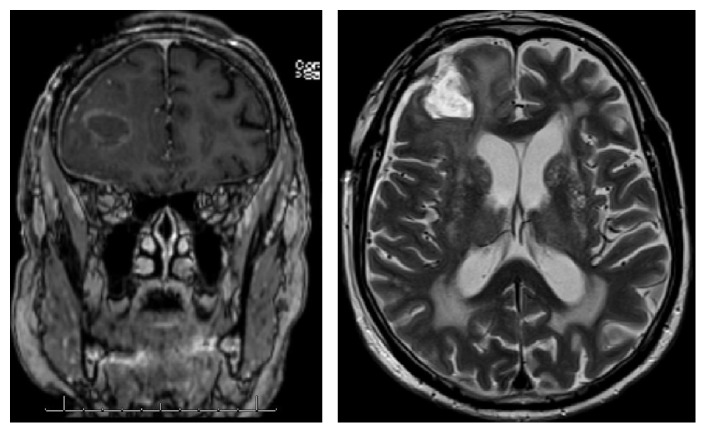
Cerebral MRI: right frontal lesion.

According to a multidisciplinary oncologic meeting, we proposed to the patient neoadjuvant chemotherapy followed by another TUR or a partial resection of the neobladder. Nevertheless, after discharge from the hospital, he presented with a seizure. A cerebral magnetic resonance imaging (Figure 4) revealed a right frontal cerebral mass, suspected as a metastatic lesion, confirmed by the biopsy-excision performed by craniotomy in the Neurosurgery Department.

A postoperative cerebral MRI revealed a residual tumor. Therefore, a stereotaxic radiotherapy of the residual tumor and resection cavity was performed. The following cerebral MRI did not show any sign of residual tumor.

Then, the patient began chemotherapy with carboplatin and gemcitabine, for the recurrence of the neobladder urothelial carcinoma. The chemotherapy was stopped after the first course because of the occurrence of a central bilateral pulmonary embolism.

**Figure 5:**
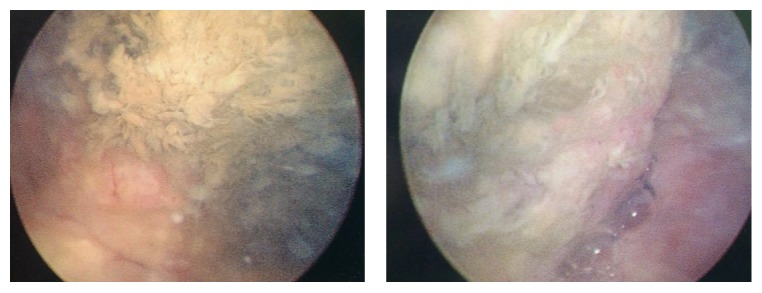
Preoperative cystoscopic views; we can note the necrotic aspect of the tumor on the anterior wall.

**Figure 6:**
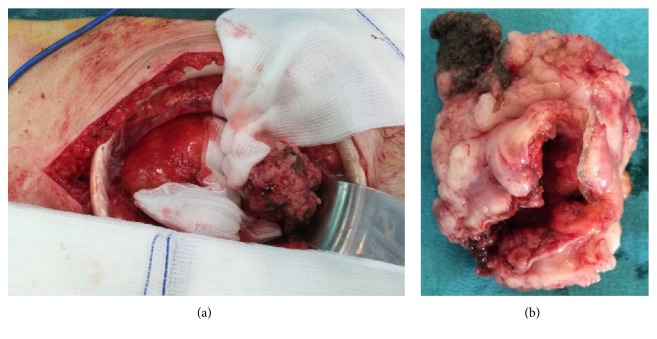
Intraoperative view (a) and resection specimen (b).

After that a cystoscopic examination was proposed to him (Figure 5), showing a necrotic tumor of the anterior wall of the neobladder, without infiltration of the opening of ureters. An anterior neobladder resection was performed immediately (Figure 6). The final histopathologic examination revealed the same high grade papillary urothelial carcinoma with malpighian differentiation, and the surgical oncological margins were free of tumor.

On the conclusion of multidisciplinary oncologic meeting, it was proposed to the patient to perform a clinical follow-up, urine cytology, and an abdominal CT-scan after three months.

## 3. Discussion

We report the case of a patient with a high grade urothelial carcinoma in an ileal neobladder nine years after radical cystoprostatectomy.

Secondary malignancy occurrence is well described in the literature, regarding ureterosigmoidostomy, cystoplasty, and intestinal conduit. The most likely presentation is adenocarcinoma [7]. Some cases of secondary adenocarcinoma developing in a replaced bowel segment of urinary diversions have been reported. Secondary adenocarcinoma developed 20 years after surgery in about 0.5% of those in whom an ileal segment was used [8]. In our knowledge, only 6 cases, including ours, are published in the literature [2–6] (Table 1). Some studies demonstrated that urothelial carcinomas have the ability to seed and implant, even on nonurothelial surfaces [1].

The Muir-Torre syndrome is a rare condition, with approximately 200 cases reported [9]. It is an autosomal dominant inherited cancer susceptibility syndrome considered to be a subset of the Lynch syndrome, also known as hereditary nonpolyposis colorectal cancer (HNPCC) syndrome [10], observed with a frequency range from 1% to 9% of HNPCC patients [11]. Its diagnosis requires one or more criteria as colorectal adenocarcinomas associated with sebaceous adenomas and/or carcinomas and genitourinary cancers. And it is confirmed by genetic analysis: the determination of the primary DNA sequence of MMR genes (MLH1, MLH2, and MSH6). Molecular genetic studies have reported microsatellite instability in the majority of samples from sebaceous and internal tumors [9–11]. According to Grignon et al., gastrointestinal tract tumors are the most frequent visceral malignancies, and urinary tract carcinomas count for 10% of the cases [12]. In this case, the patient presented with sebaceous cysts, gastrointestinal cancers, and urologic neoplasia. The Muir-Torre syndrome may have a causal role in the development of the ileal neobladder carcinoma recurrence.

## 4. Conclusion

In summary, urinary diversions using isolated gut segments bear risk of malignancy. In long-term follow-up not only adenomas and adenocarcinomas but transitional cell and papillary urothelial carcinomas may develop in the neobladder. Recurrence in the neobladder should be considered in patients with haematuria who underwent radical cystectomy and orthotopic ileal neobladder. No guidelines exist regarding the management of urothelial carcinoma recurrence. So, these patients need an expert multidisciplinary team decision.

## Figures and Tables

**Table 1:** Cases of carcinoma recurrence in an ileal neobladder.

Investigator	Age (years)	Sex	Recurrence (years)	Histology
Moore et al. [2]	62	Male	1	Papillary urothelial carcinoma
Ide et al. [3]	73	Male	11	Urothelial carcinoma in situ
Moeen [4]	59	Female	1.5	High grade invasive transitional cell carcinoma
Sánchez et al. [5]	Unknown	Unknown	Unknown	Unknown
Barba Abad et al. [6]	71	Male	8	High grade transitional cell carcinoma
Barba Abad et al. [6]	57	Male	2	Urothelial carcinoma
Present case	66	Male	9	Papillary urothelial carcinoma
